# *SIMPLEX*: simulator and postprocessor for free-electron laser experiments

**DOI:** 10.1107/S1600577515012850

**Published:** 2015-08-05

**Authors:** Takashi Tanaka

**Affiliations:** a RIKEN SPring-8 Center, Koto 1-1-1, Sayo, Hyogo 679-5148, Japan

**Keywords:** free-electron laser, simulation

## Abstract

*SIMPLEX* is a computer program developed for simulating the amplification process of free-electron lasers (FELs). It numerically solves the so-called FEL equations describing the evolution of the radiation field and growth of microbunching while the electron beam travels along the undulator. In order to reduce the numerical cost, the FEL equations have been reduced to more convenient forms for numerical implementation by applying reasonable approximations. *SIMPLEX* is equipped with a postprocessor to facilitate the retrieval of desired information from the simulation results, which is crucial for practical applications such as designing the beamline and analyzing the experimental results.

## Abstract 2

A computer program for simulating the amplification process in free-electron lasers and postprocessing the simulation results is presented.

## 1. Introduction   

In synchrotron radiation (SR) experiments, evaluating the light source performances is of great importance not only to design the beamline components but also to analyze the experimental data; several quasi-analytical formulas are available for roughly estimating the source performances of SR such as the brilliance and source size. If more detailed information is required for practical applications, numerical computations should be made to take into account all the relevant effects. This is not a trivial task for those who are not familiar with accelerator and SR physics, and thus a number of computer programs have been developed for this purpose (Walker & Diviacco, 1992 ▸; Chubar & Elleaume, 1998 ▸; Tanaka & Kitamura, 2001 ▸). This also applies to free-electron lasers (FELs), and many computer programs have been made available (Tran & Wurtele, 1989 ▸; Freund, 1995 ▸; Faatz *et al.*, 1997 ▸; Saldin *et al.*, 1999 ▸; Reiche, 1999 ▸; Fawley, 2002 ▸) to compute the light source performances of FELs.

The computation of SR is a straightforward, albeit complicated, numerical process; characteristics of radiation emitted from a single electron are first evaluated (in practice, analytical formulas can be used for this purpose in most cases), which are then convoluted with the electron distribution function. As a result, any target information can be directly computed. On the other hand, the numerical characterization of FEL radiation is inherently different from that of SR, and requires two steps: simulating and postprocessing. To be specific, the laser amplification process is simulated first with the given conditions, and then the target item is retrieved by postprocessing the simulation results.

*SIMPLEX*, which stands for the SIMulator and Postprocessor for free-electron Laser EXperiments, is a computer program to facilitate the above numerical processes necessary for evaluating the light source performances of FELs. The purpose of this paper is to introduce its capability, basic functions and available options to experimental users of FELs, the number of which is supposed to be drastically increasing because of the recent results achieved in the X-ray FEL facilities (Emma *et al.*, 2010 ▸; Ishikawa *et al.*, 2012 ▸; Allaria *et al.*, 2012 ▸).

## 2. Numerical implementation of FEL equations in *SIMPLEX*   

Before describing the details of *SIMPLEX*, let us review the basic physics behind it. In order to simulate the amplification process of FELs, we need to solve a set of equations that describe the evolution of the radiation field and motion of electrons, which are usually referred to as the FEL equations.

In solving the FEL equations, it is usually assumed that the envelope of the radiation field is a slowly varying function of the longitudinal coordinate *z* and time *t*, which is also the case in *SIMPLEX*. This makes it possible to neglect the second-order derivatives with respect to *t* and *z*, and thus significantly simplifies the equations and reduces the numerical cost.

Recently, a simulation code without this assumption has been developed (Campbell & McNeil, 2012 ▸) for special applications such as FELs taking advantage of electron beams that can violate the above condition, which are not the scope of *SIMPLEX*. Instead, we have derived mathematical formulas facilitating the numerical implementation of FEL processes with undulators having an arbitrary field distribution, which can apply to any harmonic numbers. This makes it possible not only to evaluate the effects of the undulator field errors on the FEL gain but also to describe the amplification process of FELs using exotic undulators as well as conventional ones.

In the following sections, a number of mathematical formulas for numerical implementation of FEL equations in *SIMPLEX* are presented. Note that the details of their derivations are skipped in this paper, and will be presented elsewhere.

### 2.1. Electron motion   

The motion of electrons moving in an undulator along the longitudinal (= *z*) axis is described in the six-dimensional (6-D) phase space spanned by six coordinate variables: 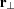, 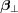, γ and ψ, where 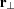 and 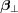 are the transverse position and relative velocity, γ is the normalized energy, and ψ denotes the longitudinal position. In simulating the FEL process, all the coordinate variables are given by solving the relevant equations at discrete longitudinal positions 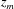 = 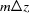, where *m* is an integer denoting the step number and 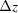 is the step interval usually given as an integer multiple of the undulator period 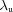.

Among the six variables, 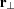 and 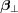 are given by solving the equation of motion of electrons moving in the magnetic field of the undulator, and can be decomposed into three terms: betatron oscillation, fast wiggling motion and trajectory wander. The first term comes from the focusing magnets and can be computed by means of the transfer matrix representing the lattice function of the undulator beamline. The other two come from the undulator field including errors and can be computed once the undulator field distribution is given. Note that the former basically vanishes when averaged over the electron distribution, while the latter are common to all the electrons if normalized by 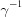.

The longitudinal position ψ satisfies the following equation,
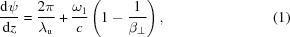
where *c* is the speed of light and 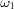 is the fundamental frequency of undulator radiation emitted from a reference electron with the average energy of 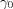. Note that ψ denotes the position relative to the reference electron in units of 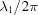 = 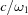 and thus is usually referred to as the electron phase.

The remaining variable γ is given by solving the equation describing the energy gain or loss of each electron moving along the undulator axis and interacting with radiation. In order to solve this energy equation, we first expand the electric field of radiation, 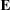, into a Fourier series with the fundamental frequency of 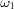, namely, 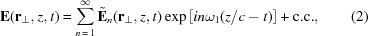
where 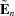 is the complex amplitude of the *n*th harmonic radiation, and is supposed to be a slowly varying function of *z* and *t*.

We then introduce a function 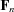 given by 
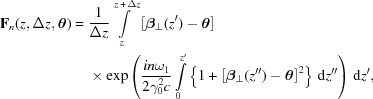
where *n* is an integer. It is worth noting that 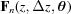 is proportional to the field amplitude of the *n*th harmonic radiation emitted by a single electron to the observation angle 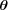, while it travels from *z* to 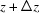 (Kim, 1985 ▸).

The electrons traveling along the undulator continuously exchange energy with radiation, which can be described by 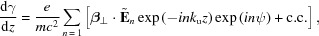
where *e* and *m* are the charge and rest mass of an electron, and 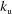 = 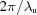. Averaging over the step interval with taking into account equation (1), we have 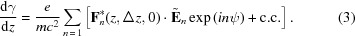
The coordinate variables of all the electrons in the electron beam are given by solving the equations described above. In most circumstances, it is not numerically effective to simulate with the real number of electrons, and thus the beamlet method (Fawley, 2002 ▸) is taken also in *SIMPLEX* to model the shot noise of the electron beam, which is the source of the self-amplified spontaneous emission (SASE) process, with much fewer macroparticles than the real number of electrons.

### 2.2. Radiation field evolution   

The evolution of the radiation field, *i.e.* how the radiation is amplified by the microbunched electron beam, can be described by the inhomogeneous wave equation derived from Maxwell’s equations given as 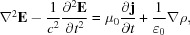
where 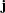 and ρ are the current and charge densities of the electron beam, 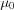 and 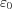 are the permeability and permittivity of vacuum, and *c* is the speed of light.

Substituting (2), neglecting the second-order derivatives with respect to *t* and *z*, multiplying 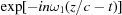 and averaging over the time duration 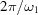, the above wave equation reduces to 
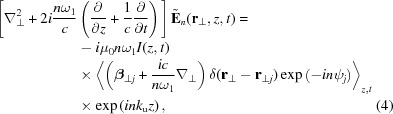
with 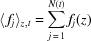
meaning that the quantity *f* is averaged over the electron distribution, where the subscript *j* indicates the *j*th electron, 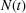 is the number of electrons contained in the time interval for averaging, and 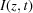 is the beam current.

In order to solve the wave equation (4), it is convenient to introduce a new independent variable 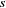 = 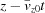 instead of *t*, where 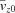 is the longitudinal velocity of the reference electron averaged over the undulator period. It is obvious that *s* refers to the relative distance with respect to the reference electron.

Let us assume that the transverse position (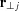) and phase (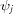) of individual electrons, and the spatial profile of 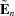, are known at the longitudinal position 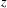 = 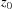. Then the radiation field at the longitudinal position 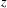 = 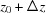 is given by a summation of two terms 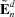 and 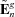, *i.e.* 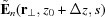 = 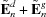, where the argument *t* has been replaced with *s* as mentioned above.

The first term 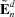 describes the diffraction of radiation, and is given by 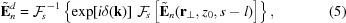
with 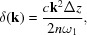
where 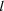 = 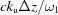 corresponds to the slippage length of radiation while the electron travels the distance 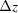 in the undulator. The operator 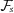 denotes the spatial Fourier transform with respect to 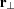, and 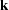 denotes the Fourier conjugate of 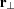.

The second term 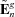 describes the growth of the radiation field, and is given by 
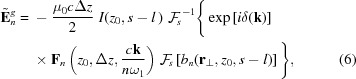
with 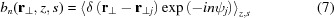
being the bunch factor density at the *n*th harmonic.

It should be noted that the function 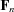 is specific to each electron because it depends on the transverse velocity 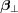. From a practical point of view, however, 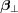 can be replaced with that of the reference electron and it is not necessary to compute 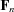 for each electron. As a result, 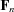 can be factored out of the averaging operation 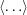 as shown in equation (6), which significantly reduces the numerical cost. This simplification also applies to the energy equation (3). In other words, 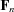 can be computed in advance as a function of *z* and 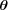 once the undulator field distribution is given.

The formulations given in equations (3) and (6), which are implemented in *SIMPLEX*, make it possible to perform FEL simulations with undulators having a periodic but arbitrary field distribution. Furthermore, it does not have to be completely periodic; the validity of the above equations may not be lost as long as it is roughly periodic so that the fundamental frequency can be defined.

### 2.3. Slicing and parallel computing   

In order to simulate the FEL process with the scheme described above, the whole electron beam is divided into a number of slices with the length of slippage *l* along the longitudinal axis, and the bunch factor 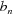 is computed for all the slices at each step 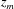. Then, the radiation field amplitude 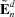 at the next step 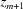 is computed using equations (5) and (6) with the slippage effect taken into account. In addition, the six coordinate variables of each macroparticle are computed with the method described in §2.1.

It is obvious that the number of slices to cover the whole electron beam can be large, leading to a significant amount of time to complete a single simulation. *SIMPLEX* is equipped with a parallel-computing option based on the message passing interface (MPI) protocol for the purpose of reducing the computation time, in which a number of numerical processes run simultaneously in parallel to solve the FEL equations for several slices at each step.

## 3. Overview of the program   

*SIMPLEX* is composed of two subprograms. One is the graphical user interface (GUI) for preprocessing the simulation conditions (§4) and postprocessing the simulation results (§6), which is built with *wxWidgets* (Smart *et al.*, 2005 ▸), the cross-platform GUI library and *OpenGL* (Shreiner *et al.*, 2013 ▸), the 2-D and 3-D graphics library. The other is the numerical code to actually perform the FEL simulation, in which the numerical methods described in §2 are implemented. Both subprograms are written in the standard C++ language, and, thanks to the portability of *wxWidgets* and *OpenGL* libraries, *SIMPLEX* can be compiled on most of the operating systems. The current version (2.1) of *SIMPLEX* is distributed only in the binary format for Microsoft Windows, Mac OS X and a couple of LINUX distributions. Note that other LINUX distributions are not supported because of the diversity of the LINUX systems. Also note that the user is requested to arrange the MPI environment, *i.e.* install MS-MPI for Microsoft Windows or MPICH for other operating systems, in order to enable the parallel computing option.

A general procedure to perform FEL simulations and retrieve desired information from the simulation results is as follows. First, the user is requested to define the simulation conditions with the assistance of the preprocessor, which invokes the numerical code with the defined conditions. Then the numerical code starts to simulate the FEL process, and creates two ASCII files and several binary files upon completion. Note that the number of binary files depends on the simulation conditions.

The parameters and options specified in the simulation are stored in one of the two ASCII files, which will be used later for postprocessing (§6). In another ASCII file, the summary of simulation results, *i.e.* several representative properties such as the pulse energy and bunch factor averaged over the whole electron beam, are stored as a function of the longitudinal position along the undulator axis.

The actual data of simulation results, *i.e.* six coordinate variables of individual macroparticles 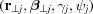, and radiation field profiles 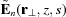, are stored in the binary files. Once these files are ready, the user can retrieve information, such as the spectrum and temporal profiles of radiation, with the assistance of the postprocessor.

## 4. Preprocessing   

Preprocessing, *i.e.* defining the numerical and boundary conditions, is an important step for any kind of simulations. In particular, FEL simulations require a huge number of input parameters and computation options to be specified by the user. Also note that the simulation results are so sensitive to some of the configurations that they should be carefully chosen or roughly optimized in advance.

**Figure 1:**
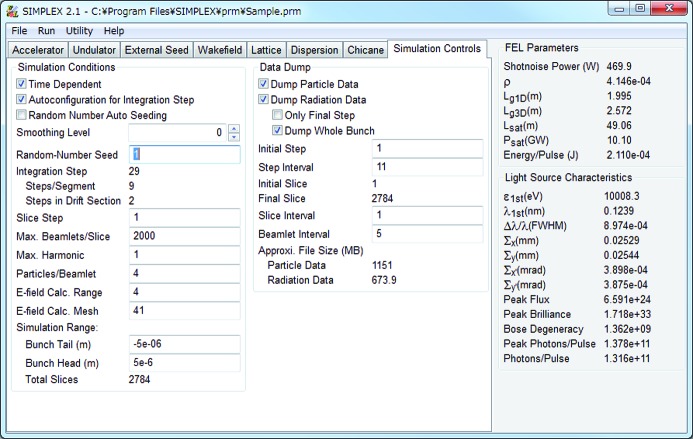
GUI as a preprocessor of *SIMPLEX*.

*SIMPLEX* is equipped with a GUI as shown in Fig. 1 ▸ to assist the preprocessing, which loads the specified parameters and options, and approximately computes the quantities relevant to the FEL process under the given conditions, so that the user can check the validity of their conditions. For example, the Pierce parameter and 1-D gain length are computed with well known analytical formulas (Bonifacio *et al.*, 1984 ▸), while the 3-D gain length, saturation length and saturation power are computed with empirical formulas (Xie, 2000 ▸). In addition, light source performances such as the source size, angular divergence, bandwidth, photon flux and brightness are roughly estimated based on the assumption that the FEL radiation is spatially coherent.

The parameters and options required for FEL simulations in *SIMPLEX* are categorized into three groups: specifications of the FEL system, options to evaluate the effect of various errors, and numerical and boundary conditions to carry out the simulation.

The first group specifies the core parameters of the FEL system, such as the electron beam, undulator, lattice function and seed light if necessary. In addition to standard Gaussian profiles, it is also possible to specify the electron beam by loading the 6-D macroparticle data, which may be probably generated by other programs. Note that *SIMPLEX* preprocessor offers a function to retrieve the relevant parameters such as the current, energy, slice emittance and slice energy spread as a function of the bunch position, which is useful to check as to whether the format of the input data file is valid. As for the undulator specifications, the device type as well as its standard parameters can be chosen from four possible options: linear, helical, elliptical and multi-harmonic undulators, where the latter denotes an exotic undulator containing multiple harmonic components with the fundamental period of 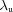.

The second group refers to a number of options to evaluate the effects of possible errors degrading the FEL gain, such as the field errors of the undulator including its misalignment, and the trajectory errors coming from the misaligned quadrupole magnets and beam position monitors.

The third group defines the numerical conditions, such as the step interval, numbers of macroparticles and beamlets, spatial range and grid points to evaluate the radiation field, and settings for the random number generator. Configurations for parallel computing and exporting the simulation results also need to be specified.

Besides the parameter input and option selection described above, the *SIMPLEX* preprocessor also offers a function to check the specified configurations graphically, *i.e.* to create a graphical plot that is relevant to specific configurations such as the phase error and trajectory wander to check the field error of the target undulator segment, betatron function along the undulator beamline to check the arrangement of the focusing magnets, and dispersion function in the presence of misaligned components to check the effects of the misalignment.

## 5. Options for single-pass FELs   

*SIMPLEX* is equipped with a number of options and functions useful for simulating the single-pass FEL, the main FEL scheme currently available in short-wavelength regions, which are explained in the following sections.

### 5.1. Self-seeding   

Self-seeding (Feldhaus *et al.*, 1997 ▸) is one of the seeding schemes for short-wavelength FELs, in which the undulator is divided into two sections, and the SASE radiation generated in the first half is monochromated by a monochromator before entering the second half, and effectively works as a coherent seed light. *SIMPLEX* is equipped with two options necessary to simulate the self-seeding process: creating a magnetic chicane and inserting a monochromator.

#### 5.1.1. Magnetic chicane   

This is an option to replace one of the undulator segments with a magnetic chicane composed of four dipole magnets, which makes it possible to retard the electron beam with respect to the radiation. This option can be also used to simulate the pulse length measurement by means of the autocorrelation technique (Geloni *et al.*, 2010 ▸) as well as the self-seeding.

#### 5.1.2. Monochromator   

This is an option to insert a monochromator in the magnetic chicane described above, in order to monochromate the SASE radiation generated in the first half. In addition to typical monochromator schemes (geometry and crystal) for self-seeding available in the X-ray region, including the forward Bragg diffraction (Geloni *et al.*, 2011 ▸), the user can specify any monochromator by importing the numerical data to specify the complex reflectance (or transmittance) of the monochromator.

### 5.2. Redistributing the macroparticles   

When the microbunched electron beam passes through a magnetic chicane having a large momentum compaction, the microbunch is usually washed out because the large energy modulation works to smear the density modulation. In FEL simulations based on the beamlet method like *SIMPLEX*, this phenomena cannot be accurately described because of the finite number of macroparticles and beamlets. This option tries to redistribute the macroparticles to reproduce the bunch factor after the chicane, based on analytical formulas.

### 5.3. Seeking the betatron matching condition   

In the undulator beamline where a number of undulator segments are regularly spaced and focusing magnets are placed in between, the betatron function can be made to be periodic with small deviations, if the initial Twiss parameters are properly optimized, which is usually referred to as the betatron matching. This in turn means that the betatron function can diverge, or at least be modulated, if the betatron matching condition is not satisfied. The *SIMPLEX* preprocessor offers a function to compute the optimum values of the initial Twiss parameters for a given layout of focusing magnets, so that the user does not have to manually configure these parameters.

### 5.4. Evaluating the wakefield effects   

The wakefield is an electromagnetic field induced by interaction between electrons and the surrounding environment, and gives rise to a correlated energy variation along the electron beam, which eventually leads to a FEL gain reduction. *SIMPLEX* is equipped with an option to compute the wakefield-induced energy variation while the electron beam travels along the undulator.

## 6. Postprocessing   

After completing the simulations, the user can retrieve desired information from the files that store the simulation results. As mentioned before, the macroparticle distribution 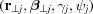 and radiation field profile 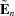 are stored in several binary files, which will be processed and visualized with the assistance of the postprocessor.

### 6.1. Radiation field   

Retrieving the radiation field profile 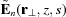 followed by a number of numerical operations gives important information on light source performances such as the radiation power and photon flux. *SIMPLEX* offers many functions to visualize these items in terms of temporal, spectral, spatial and angular profiles, which are given as a function of the step, slice and photon energy. This makes it possible to graphically investigate the amplification process in FELs, such as the evolution of radiation power, spectral narrowing and optical guiding. If the simulation is performed with undulators other than conventional ones (linear and helical undulators), the radiation field 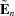 is computed as a vector but not as a scalar, and thus the Stokes parameters can be also retrieved for the purpose of investigating the polarization property of radiation.

In addition to the standard postprocessing methods described above, *SIMPLEX* offers an option to analyze the radiation properties based on the Wigner function method (Wigner, 1932 ▸). To be specific, the photon density profile in the transverse phase space 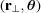 is computed using the definition of the Wigner function, 
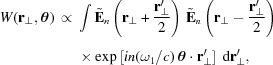
where other arguments *z* and *s* are omitted for simplicity. Note that the above Wigner function is actually projected onto the horizontal or vertical phase spaces in *SIMPLEX* to facilitate visualization. The photon density profile in the longitudinal phase space 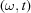 is also available using the similar definition.

It is worth mentioning that supplemental information can be computed once the above profiles are retrieved from the simulation results; for example, the beam size and angular divergence are computed from the spatial and angular profiles, the pulse length and bandwidth are computed from the temporal and spectral profiles, and the overall degree of spatial coherence (Bastiaans, 1986 ▸) is computed from the transverse Wigner function. *SIMPLEX* offers an option to show and/or save these numerical data if available.

### 6.2. Macroparticle   

The most straightforward way to visualize the macroparticle distribution is to trace their motion in the phase space while the electron beam travels along the undulator. This enables one to investigate how the energy and density modulations are induced and how the microbunch evolves.

In addition to the above method, *SIMPLEX* offers two functions to process the macroparticle distribution. One is to retrieve the bunch factor 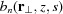 using equation (7) and visualize its spatial and temporal profiles. The other is to compute the electron beam properties, such as the energy variation, energy spread and beam current at respective slices, and visualize their temporal profiles.

## 7. Examples   

Now let us show several examples to introduce the capability and available functions of *SIMPLEX* as a computer program to evaluate the light source performances of FELs. Table 1 ▸ summarizes common parameters assumed in the examples, which correspond to those found by Ishikawa *et al.* (2012 ▸), reporting the first lasing of the SPring-8 Angstrom Compact Free-Electron Laser (SACLA). Note that the laser performance available in SACLA has been considerably improved since then, in terms of the pulse energy and gain length, suggesting that the actual beam parameters currently realised in SACLA are much better.

### 7.1. Gain curves for different undulator configurations   

One of the important features of *SIMPLEX* is the flexibility of acceptable undulator configurations. To be specific, undulators with an arbitrary field distribution are accepted thanks to the formulation of FEL equations presented in §2. As an example, let us make a comparison between undulator types and investigate the effects due to undulator field errors in terms of the gain curve, *i.e.* the FEL pulse energy plotted as a function of the longitudinal coordinate *z*.

**Figure 2:**
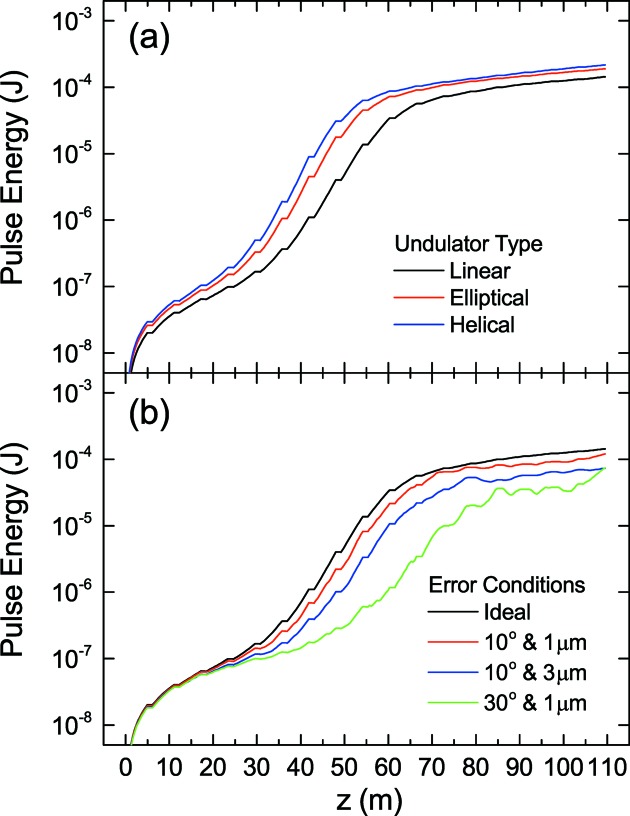
Gain curves for different undulator configurations. (*a*) Comparison between different undulator types. (*b*) Effects due to the undulator field error in terms of the phase error and trajectory wander.

Fig. 2(*a*) ▸ shows a comparison between three different undulator types: linear, elliptical and helical. Note that the effective deflection parameter defined by 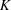 = 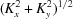 has been kept constant for each case, where 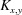 are the deflection parameters corresponding to the horizontal and vertical field components, respectively, and 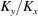 = 2 has been assumed for the elliptical undulator.

It is well known that the FEL performance with the helical undulator is better than that with the linear undulator, in terms of the gain length and saturation power. Thus, the performance with the elliptical undulator is expected to be reasonably between the two undulator types according to 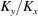. The results shown in Fig. 2(*a*) ▸ are actually consistent with the above discussion.

Fig. 2(*b*) ▸ shows the effects due to the undulator field errors, in terms of the phase error 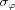 and trajectory wander 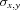 in both directions. Note that the *SIMPLEX* preprocessor offers a function to generate undulator field errors to reproduce the values of 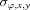 specified by the user. In this example, three different conditions have been examined: 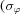, 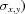 = (10°, 1 µm), (10°, 3 µm) and (30°, 1 µm). We find that the FEL performance is more sensitive to the increase in 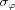 than 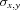 in this example. Note, however, that the above discussion may not be universal and depends on the parameters and conditions.

### 7.2. Temporal profiles of radiation power and bunch factor   

**Figure 3:**
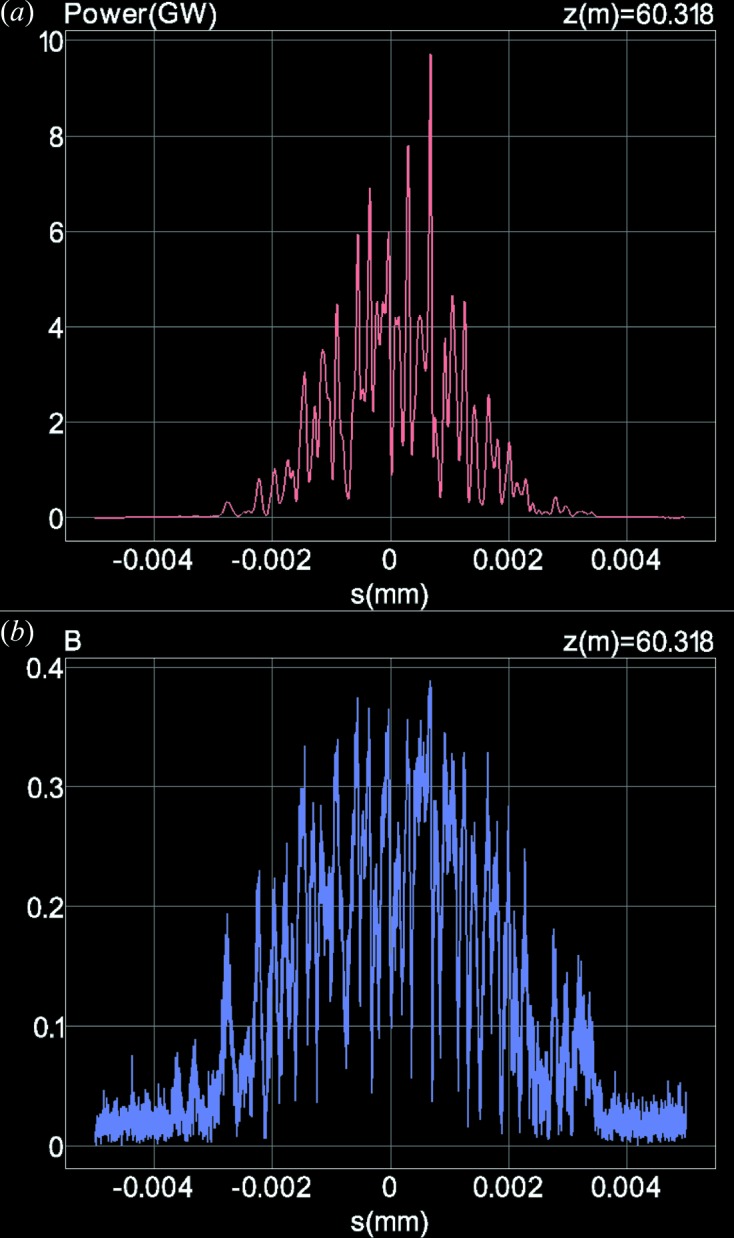
Temporal profiles of (*a*) radiation power and (*b*) bunch factor at the longitudinal position of 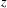 = 60.3 m.

The *SIMPLEX* postprocessor can visualize the simulation results as an animation by sweeping a relevant variable such as the step, slice or photon energy. One example is shown in Fig. 3 ▸, in which the temporal profiles of the radiation power and bunch factor computed by integrating over 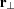 are plotted as a function of *s* at a longitudinal position of *z* = 60.3 m, where the pulse energy almost saturates. Note that this plot just shows a captured image of an animation illustrating the evolution of the temporal profiles while the electron travels along the undulator, which is created by sweeping the step number.

### 7.3. Transverse Wigner functions   

**Figure 4:**
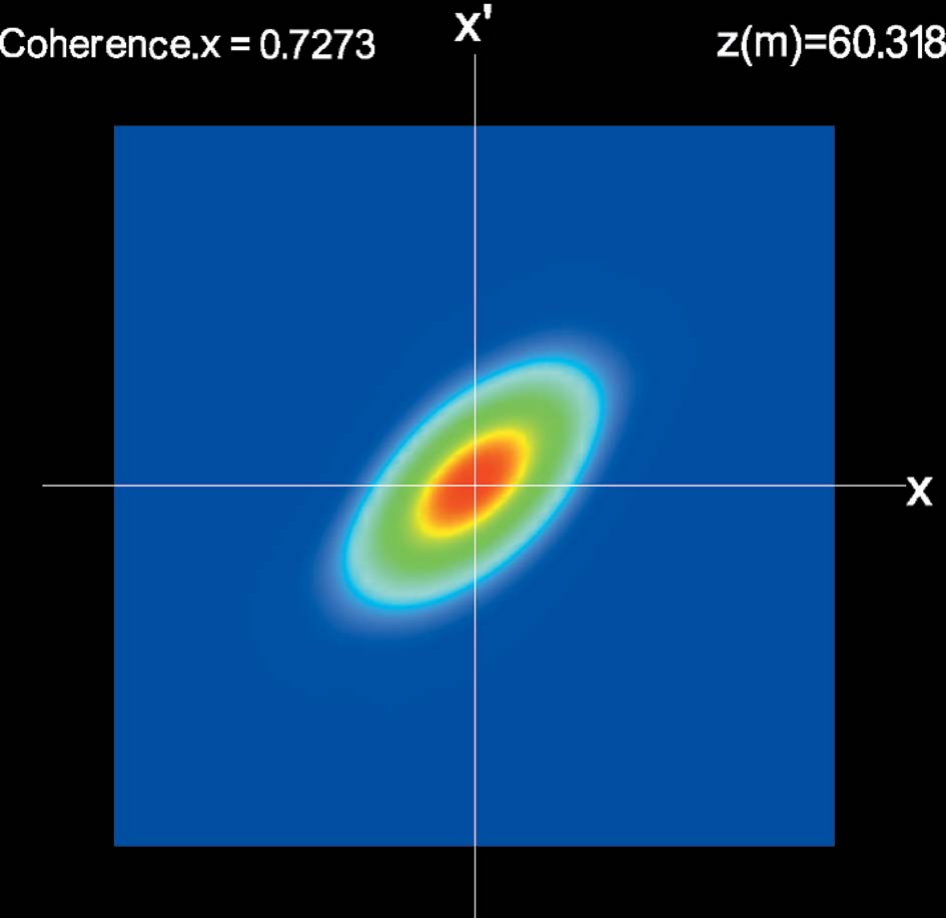
Transverse Wigner function averaged over all the slices and projected on 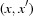 phase space at the longitudinal position of 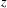 = 60.3 m.

Analysis based on the Winger function is useful especially for investigating the transverse properties of FEL radiation. Fig. 4 ▸ shows the transverse Wigner function at *z* = 60.3 m, which is projected on the horizontal phase space 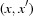 and averaged over the whole electron beam. We find that the photon beam is diverging and thus the source point is located slightly upstream. Also note that the overall degree of spatial coherence, which can be computed from the Wigner function, is indicated.

### 7.4. Self-seeding   

**Figure 5:**
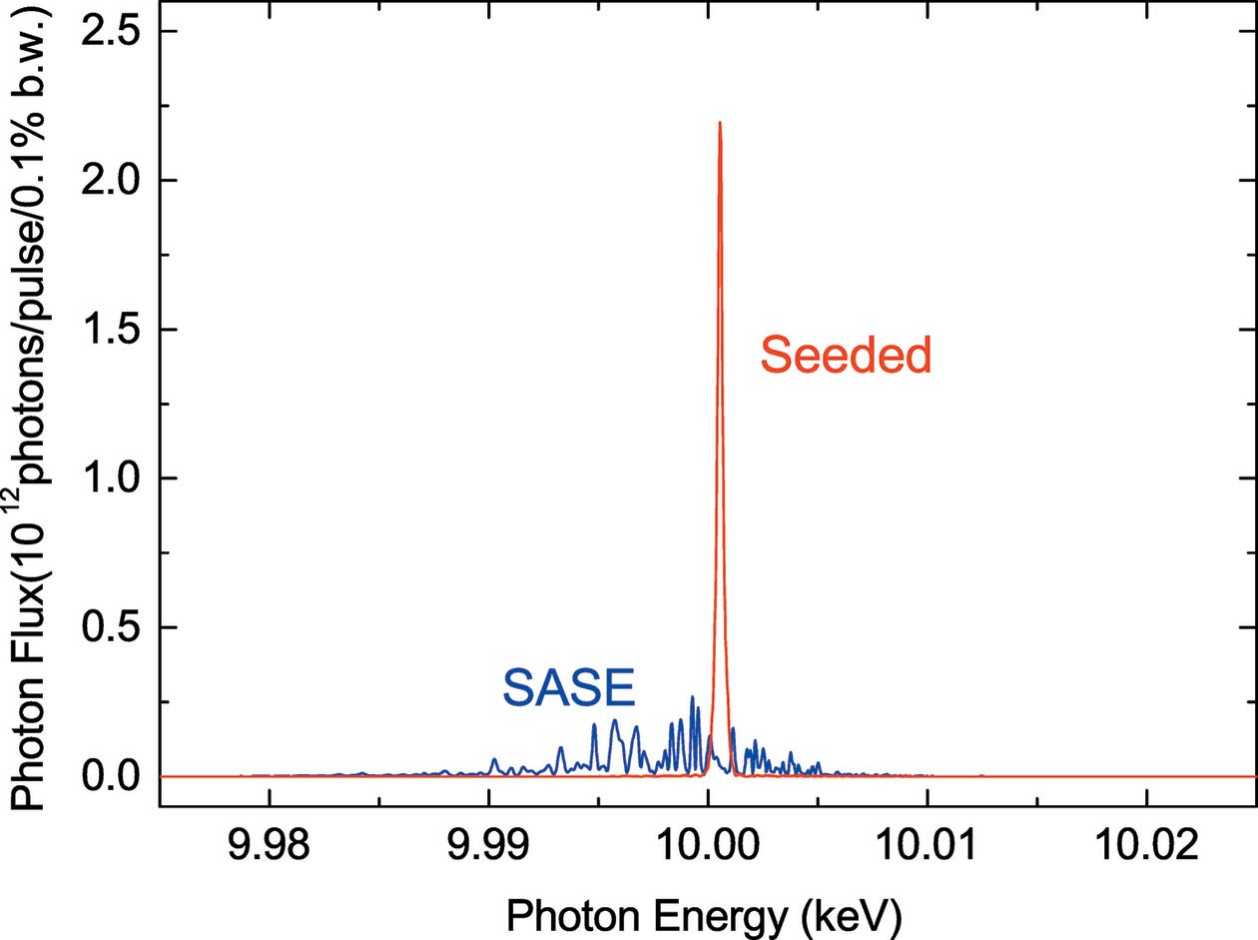
Comparison of spectra with (seeded) and without (SASE) the self-seeding chicane.

The last example demonstrates the spectral improvement by means of the self-seeding scheme based on the forward Bragg diffraction. We have assumed that a magnetic chicane is installed in replacement of the ninth undulator segment, where a diamond monocrystal of thickness 0.18 mm is inserted with the (400) Bragg diffraction geometry. Fig. 5 ▸ shows a comparison of spectra with and without the self-seeding chicane, which well quantifies the spectral difference between the SASE and seeded FELs.

## 8. Summary   

Besides those presented in this paper, *SIMPLEX* is equipped with many other functions and options to facilitate the FEL simulations with more practical conditions, such as loading the numerical data describing the temporal profiles of sliced beam parameters (current, mean energy, energy spread, *etc.*), and importing the undulator field distribution actually measured with a field measurement apparatus. For more specialized applications such as the HGHG (high-gain harmonic generation) FELs, the results of a previous simulation can be loaded to be recursively used in another simulation.

Benchmarking of *SIMPLEX* was carried out by comparison with *GENESIS* (Reiche, 1999 ▸), one of the most acknowledged FEL simulation codes, in both the time-dependent and steady-state simulation modes. The agreement was found to be fairly good in terms of the gain length and saturation power.

*SIMPLEX* is freely available from the Web (Tanaka, 2015 ▸). It should be emphasized that *SIMPLEX* does not require any other commercial software or libraries for preprocessing, simulating and postprocessing, except the MPI environment for parallel computing if necessary, which is also available online freely.

## Figures and Tables

**Table 1:** Parameters assumed in the simulation example

	Value	Unit
Electron beam parameter		
Electron energy	7.05	GeV
Bunch length (r.m.s.)	2.5	µm
Bunch charge	0.075	nC
Normalized emittance	0.7	mm mrad
Energy spread	10^−4^	
Average betatron function	25	m

Undulator parameter		
Deflection parameter (*K*)	1.8	
Period length	18	mm
Segment length	5	m
Drift length	1.15	m
Number of segments	18	
Fundamental photon energy	10	keV
